# Biodegradation Study of Styrene–Butadiene Composites with Incorporated *Arthrospira platensis* Biomass

**DOI:** 10.3390/polym16091218

**Published:** 2024-04-26

**Authors:** Marius Bumbac, Cristina Mihaela Nicolescu, Traian Zaharescu, Ion Valentin Gurgu, Costel Bumbac, Elena Elisabeta Manea, Ioana Alexandra Ionescu, Bogdan-Catalin Serban, Octavian Buiu, Crinela Dumitrescu

**Affiliations:** 1 Faculty of Science and Arts, Valahia University of Targoviste, 13 Aleea Sinaia, 130004 Targoviste, Dambovita, Romania; marius.bumbac@valahia.ro (M.B.); crinela.dumitrescu@valahia.ro (C.D.); 2 Institute of Multidisciplinary Research for Science and Technology, Valahia University of Targoviste, 13 Aleea Sinaia, 130004 Targoviste, Dambovita, Romania; traian_zaharescu@yahoo.com (T.Z.); valentin.gurgu@valahia.ro (I.V.G.); 3 National Institute for Electrical Engineering, Advanced Research (INCDIE ICPE CA), 313 Splaiul Unirii, 030138 Bucharest, Ilfov, Romania; 4 National Research and Development Institute for Industrial Ecology-ECOIND, 57-73 Drumul Podu Dambovitei, District 6, 060652 Bucharest, Ilfov, Romania; costel.bumbac@incdecoind.ro (C.B.); elena.manea@incdecoind.ro (E.E.M.); ioana.ionescu@incdecoind.ro (I.A.I.); 5 IMT Bucharest, National Institute for Research and Development in Microtechnologies, 126A Erou Iancu Nicolae, 077190 Voluntari, Ilfov, Romania; octavian.buiu@imt.ro

**Keywords:** microalgae biomass, *Arthrospira platensis*, styrene–butadiene rubber, biodegradation, polymer bio-composites

## Abstract

The preparation of polymer composites that incorporate material of a biogenic nature in the polymer matrices may lead to a reduction in fossil polymer consumption and a potentially higher biodegradability. Furthermore, microalgae biomass as biogenic filler has the advantage of fast growth and high tolerance to different types of culture media with higher production yields than those provided by the biomass of terrestrial crops. On the other hand, algal biomass can be a secondary product in wastewater treatment processes. For the present study, an SBS polymer composite (SBSC) containing 25% (*w*/*w*) copolymer SBS1 (linear copolymer: 30% styrene and 70% butadiene), 50% (*w*/*w*) copolymer SBS2 (linear copolymer: 40% styrene and 60% butadiene), and 25% (*w*/*w*) paraffin oil was prepared. Arthrospira platensis biomass (moisture content 6.0 ± 0.5%) was incorporated into the SBSC in 5, 10, 20, and 30% (*w*/*w*) ratios to obtain polymer composites with spirulina biomass. For the biodegradation studies, the ISO 14855-1:2012(E) standard was applied, with slight changes, as per the specificity of our experiments. The degradation of the studied materials was followed by quantitatively monitoring the CO_2_ resulting from the degradation process and captured by absorption in NaOH solution 0.5 mol/L. The structural and morphological changes induced by the industrial composting test on the materials were followed by physical–mechanical, FTIR, SEM, and DSC analysis. The obtained results were compared to create a picture of the material transformation during the composting period. Thus, the collected data indicate two biodegradation processes, of the polymer and the biomass, which take place at the same time at different rates, which influence each other. On the other hand, it is found that the material becomes less ordered, with a sponge-like morphology; the increase in the percentage of biomass leads to an advanced degree of degradation of the material. The FTIR analysis data suggest the possibility of the formation of peptide bonds between the aromatic nuclei in the styrene block and the molecular residues resulting from biomass biodegradation. It seems that in industrial composting conditions, the area of the polystyrene blocks from the SBS-based composite is preferentially transformed in the process.

## 1. Introduction

The widely accepted standards for full biodegradability of a polymer should refer to the complete conversion by microorganisms into carbon dioxide, water, minerals, and biomass. The biodegradation process should occur under relevant environmental conditions and timeframes, without causing harm to the environment or leaving behind toxic residues [1,2,3,4]. Biodegradable polymers may be naturally occurring or may be synthesized by chemical means. Following this reasoning, biodegradable polymers may be classified into the following three categories:

- *Natural polymers* occur naturally in various sources such as plants, animals, and microorganisms. Examples include starch, cellulose, proteins, and poly-β-hydroxybutyrate (PHB). These polymers are inherently biodegradable and can be broken down by microorganisms in the environment [5,6,7].
- *Modified natural polymers* obtained from natural polymers can be chemically or biologically modified to enhance their properties or biodegradability. For example, cellulose can be modified to cellulose acetate or lignocellulose esters. Polyalkanoate copolymers are also examples of chemically modified natural polymers. These modifications may alter the degradation rate or other characteristics of the polymers [8,9,10].
- *Synthetic biodegradable polymers* are modified by incorporating natural biodegradable components. This involves blending or complexing the synthetic polymer with materials such as starch, reclaimed cellulose, or natural rubber. The addition of these natural components aims to improve the biodegradability of the synthetic polymer by providing biodegradable elements that microorganisms can break down [5,11,12,13,14].

Biodegradation requires carbon, energy, and electrons as essential resources for microbial metabolism. Microorganisms require a source of carbon to build their cellular components, energy to fuel their metabolic processes, and electrons to drive redox reactions. There are specific terms associated with the source of each of these items, which can help define and categorize organisms based on their metabolic strategies [15]. The approach to enhance the biodegradability of a polymer composite is to incorporate biomass that contains proteins and polysaccharides [16]. These organic components can serve as nutrients for microorganisms, facilitating their growth and metabolic activity. At the life end of polymers, the material is recycled or ends up in a landfill or other environment where the microorganisms existing in the surrounding soil or compost may colonize the material. These microorganisms, such as bacteria or fungi, utilize the proteins and polysaccharides from the biomass as a food source. As a result, they may prove efficient in breaking down the polymer matrix, leading to its degradation. By providing the necessary nutrients to the microorganisms, the added biomass promotes the growth of microbial populations, enhancing the overall biodegradation process. As the microorganisms metabolize the organic components, they may produce enzymes that can initiate the breakdown of the polymer chains, ultimately resulting in the complete degradation of the composite [6,17]. The success of this approach depends on factors such as the composition and structure of the polymer composite, the specific microorganisms present in the environment, and the availability of suitable conditions for biodegradation (e.g., moisture, temperature, oxygen levels, pH). Additionally, the amount and type of biomass added to the composite should be carefully optimized to achieve the desired biodegradability while maintaining the mechanical and functional properties of the material.

Spirulina (*Arthrospira platensis*) is a suitable candidate as a biomass filler for developing polymer materials with enhanced biodegradability. It is a type of cyanobacteria, often referred to as blue-green algae, and it has gained attention in the last decades for its various potential applications, including the use as a biomass filler in biodegradable polymers with natural rubber [18], ethylene propylene diene monomer rubber (EPDM) [19], polylactic acid (PLA) [20], poly(butylene succinate) (PBS) [21], polyvinyl alcohol (PVA) [22], and polypropylene (PP) [23]. 

Spirulina biomass presents premises for abundance and sustainability as it is available from large-scale farms with a rapid growth rate. The main components of *Arthrospira platensis* biomass are carbohydrates (10–20%), lipids (5–10%), proteins (55–70%), and pigments. As may be noticed, this composition indicates spirulina biomass as a high-protein-content biogenic material for further use in polymer composites [24]. 

The successful incorporation of spirulina biomass into polymer materials requires careful consideration of factors such as processing methods, compatibility with the polymer matrix, and optimization of the spirulina content to achieve desired properties without compromising the overall performance of the composite material. Spirulina biomass shows promise as a biomass filler for developing biodegradable polymer materials, contributing to the goal of reducing the environmental impact of plastics and promoting sustainable alternatives.

Poly(styrene–butadiene–styrene) copolymers (SBS) are hard rubbers used for elements capable of maintaining their tensile shape, as they are wear-resistant with low biodegradability. SBS polymers consist of glassy polystyrene domains connected by polybutadiene segments, thus having a two-phase morphology.

The present paper reports on the study of synthetic polymers incorporating spirulina biomass into a styrene–butadiene–styrene (SBS) polymer matrix. Thus, a composite obtained by mixing pristine SBS and paraffin oil (3:1, *w*/*w*) was used to incorporate *Arthrospira platensis* biomass in 5, 10, 20, and 30% (*w*/*w*) ratios. The polymer composites, then subjected to the industrial composting degradation procedure, were characterized and compared, aiming to evaluate the effect of algal biomass addition to the polymer material characteristics while biodegrading in the experimental conditions.

## 2. Materials and Methods

### 2.1. Polymer Bio-Composites’ Preparation

The composition choice for the studied polymer composites, as well as the preparation procedure, was recently reported by the authors [16]. Thus, the mass % composition for the base polymer mixture employed was 25% linear SBS copolymer (with 30% styrene and 70% butadiene, LCY Grit Corporation, Taiwan), 50% branched SBS copolymer (with 40% styrene and 60% butadiene, LCY Grit Corporation, Kaohsiung City, Taiwan), and 25% paraffin oil (Apar Ltd., Mumbai, India). The biogenic content in the final bio-composite polymers was 5, 10, 20, and 30% (*w*/*w*). Microalgal biomass in powder form with 5.8 ± 0.5% (*w*/*w*) moisture, measured at the moment of extrusion, was purchased from Hyperici Pharm SRL, Targoviste, Romania.

### 2.2. Experimental Setup for the Biodegradation Testing (Industrial Composting Conditions)

**Figure 1:**
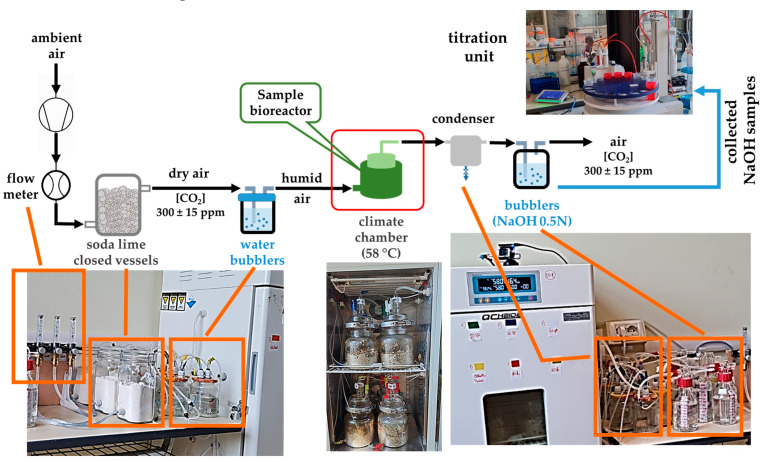
Laboratory biodegradation testing installation.

The installation for testing the biodegradability of the studied polymer composites was established considering both the provisions of the ISO 14855-1/2022 (58 °C, 50% humidity) [25], as well as some practical aspects in conducting experiments in the laboratory. As may be observed in Figure 1, the components of this installation were (*i*) a *gas inlet circuit* (CO_2_ free air), (*ii*) *climatic chambers* (Haida International, Dongguan City, China), (*iii*) *bioreactors* for samples made of cylindrical glass vessels tightly covered with customized design (Adrian Sistem SRL, Bucharest, Romania) that were placed in the climatic chambers, and (*iv*) a *gas outlet circuit* (with CO_2_ resulting from the biodegradation processes). 

Each bioreactor contained an individual sample, with 100 g sample, together with 600 g vermiculite (dry weight/d.w.) that was previously activated in our laboratories, according to ISO procedure [25], as mentioned above (using compost extract and nutritive synthetic solution). The technical balance used for the weighing operations was PCB 3500-2 (Kern & Sohn GmbH, Balingen, Germany). For each testing series, one blank bioreactor (containing vermiculite only) and two control bioreactors (one containing activated vermiculite with cellulose as the positive control sample and the other with polyethylene as the negative control sample) were used. The bioreactors were checked weekly for humidity by overall weighing, and the 50 ± 2% value was re-established, where needed, by adding deionized water (<0.1 μS·cm^−1^ at 25 °C). 

The inlet circuit contained a compressor KA5-ESM5 and an air dryer EDX9-CC1030394 (Garden Denver, Quincy, IL, USA) that allowed the CO_2_-free air to enter each bioreactor via flowmeters at 2 L/min. Before entering the bioreactors, the inlet air was first passed through soda lime glass vessels, then through water bubblers filled with deionized H_2_O to slightly increase the humidity.

The outlet circuit, corresponding to the evolved gases during sample biodegradation processes, contained silicon tubes with unidirectional valves towards 500 mL glass gas-washers filled with NaOH 0.5 M solution for CO_2_ trapping, via decanters that allowed undesirable dilutions of the trapping solutions because of entrained water vapors. The decrease in the hydroxide concentration (correlated with the CO_2_ evolved in each bioreactor) was monitored by automatic potentiometric titration with a DGi111-SC combined glass electrode connected to a T5–Excellence titrator with InMotion Flex autosampler (Mettler Toledo, Greinfensee, Switzerland), using HCl 0.1 M solution as titrant. Samples of CO_2_-trapping sodium hydroxide solutions were periodically collected (10 mL, triplicate measurements) from gas washers and titrated. The result from titration was also an indication of whether refreshing the NaOH 0.5 M was necessary or not so that a reasonable quantity of hydroxide is available to react with potentially further CO_2_ formed through each sample biodegradation in the studied conditions. Final calculations of CO_2_ evolved from each vessel considered the titer of each fresh NaOH 0.5 M solution, the titer of HCl 0.1 M titrant solutions, and the unknown concentration hydroxide volumes sampled from gas-washers. 

Apart from the blank sample and the positive and negative control samples, the SBS-based polymer matrix composite was also considered a control sample and received the codification SBSC. Tested polymer bio-composite samples containing spirulina biomass at 5, 10, 20, and 30% (*w*/*w*) received the codes Sp5SBS, Sp10SBS, Sp20SBS, and Sp30SBS, respectively. 

### 2.3. Characterization of Structure and Morphology of Tested Bio-Composites

▪ Physical and mechanical testing

Potential changes in the specific gravity and flowability of the studied samples, after 100 days and 180 days of biodegradation, respectively, in industrial composting conditions, were monitored. Eprouvettes prepared according to ISO 291:2008 [26] were tested for the melting flow index (MFI) with a specialized tester (Haida Plastic Melt Flow Index Testing Machine, Dongguan, China), while the VF4601 density kit mounted on a Secura225D-1CEU balance (Sartorius, Göttingen, Germany) was used to measure the specific gravity.

▪ Fourier Transform Infrared spectroscopy (FTIR)

Infrared spectroscopy (FTIR) was applied in the reported study to evaluate changes that may appear in the molecular structure of bio-composites as an effect of biodegradation processes after 100 days and after 180 days. Comparisons with the base polymer matrix behavior in the same conditions were made. 

Data were recorded on a Vertex 80 infrared spectrometer (Bruker, Karlsruhe, Germany) with ATR (attenuated total reflectance) sample system, and thus, solid samples were used with no specific preparation. The scanning wavenumber range was 4000 cm^−1^ to 400 cm^−1^, at a spectral resolution of 4 cm^−1^, and 32 scans per sample were recorded. Pristine composites (base polymer matrix SBSC and the bio-composites with incorporated spirulina biomass) and products after the two studied biodegradation time periods were scanned.

▪ Scanning Electronic Microscopy (SEM)

The electronic scanning microscopy system SEM-Quanta FEG 250 (Thermo Fischer Scientific, Waltham, MA, USA) in secondary electron mode with an Everhart–Thornley Detector was used to conduct morphological investigations of composite polymers containing algal biomass in different mass ratios, after biodegradation. Freshly cut samples with a 2 mm thickness were mounted on microscopy stubs, after they were fully covered with carbon bands, specific for SEM measurements. Comparisons of images collected for bio-composite eprouvettes after 100 and 180 days of aerobic biodegradation tests with their initial surface condition were made and discussed.

▪ Thermal Analysis—Differential Scanning Calorimetry (DSC)

The thermal analysis system DSC3-StarE (software version SW 17.00) (Mettler Toledo, Greinfensee, Switzerland) was used to record DSC thermograms of polymer bio-composites and control samples (base polymer matrix). Tests were run in the temperature range of 30 to 300 °C, at a heating rate of 10 °C/min, on samples with a mass of 10 ± 1 mg, weighed on a Secura225D-1CEU (Sartorius, Göttingen, Germany) analytical balance with a precision of 1 × 10^−5^ g, placed in standard aluminum pans of 40 µL. In the DSC measurement cell, an empty 40 µL aluminum pan was used as a reference.

## 3. Results

### 3.1. Biodegradation Testing (Industrial Composting)

**Figure 2:**
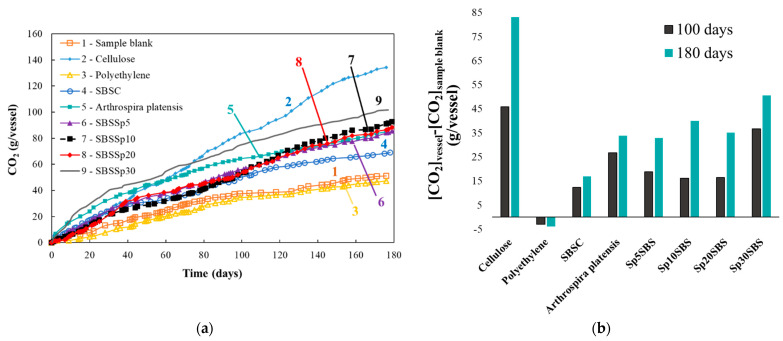
Evolution of CO_2_ mass (g/vessel) trapped in the NaOH 0.5N solution in the industrial composting test: (**a**) comparative evolution of trapped CO_2_ in time and (**b**) calculated difference between the mass of CO_2_ trapped in the bioreactors with samples and the sample blank.

The total mass of CO_2_ that was absorbed in the bubblers with NaOH 0.5 M solution is presented in Figure 2a and was calculated according to Equation (2), where ${m_{{CO}_{2}}}_{i}$ is the carbon dioxide mass calculated according to Equation (1) between two titration points, “*i*” and “*i* + 1”. The values presented for $c_{i}$ and $c_{i+1}$ represent the concentrations of sodium hydroxide solutions at the titration points “*i*” and “*i* + 1”, whereas the volumes $V_{i}$ and $V_{i+1}$ are the volumes of solutions in bubblers before titration.

(1) $${m_{{CO}_{2}}}_{i+1}={m_{{CO}_{2}}}_{i}+\left(c_{i}\cdot V_{i}-c_{i+1}\cdot V_{i+1}\right)$$

(2) $${m_{{CO}_{2}}}_{total}=\sum_{i=1}^{n}{m_{{CO}_{2}}}_{i}$$

(3) $${m_{{CO}_{2}}}_{composting}={m_{{CO}_{2}}}_{vessel}-{m_{{CO}_{2}}}_{sample\;blank}$$

The difference between the CO_2_ mass trapped in the bubblers with NaOH 0.5 M solution from the bioreactors with samples and the CO_2_ trapped from the sample blank vessel is denoted as ${m_{{CO}_{2}}}_{composting}$ (Equation (3)) and is presented in Figure 2b for 100 days and 180 days of industrial composting testing. The cellulose bioreactor (positive control) produced the highest amount of CO_2_ absorbed in the bubblers compared with the other samples (Figure 2a,b).

The variation in the measured mass of CO_2_ for the SBSC composite shows an almost linear increase followed by an inflection point at approximately 100 days. The same type of evolution is recorded for the blank sample (which only contains the culture medium) and for the negative sample (polyethylene). The graphical representation of ${m_{{CO}_{2}}}_{composting}$ (Figure 2b) showed that the polymer under test showed significant degradation compared to polyethylene, suggesting that the studied SBS-based polymers have a certain level of biodegradability.

Also notable in Figure 2 is the behavior of the spirulina biomass, which shows an increase in the mass of CO_2_ evolved in the first 100 days, followed by a decrease in the rate of CO_2_ production. This evolution may be assigned to the unique characteristic of spirulina biomass, which has a high protein content (40–70%) [27] compared to other types of plant biomass. The spirulina biomass produces more carbon dioxide in the first 100 days, but after this period, the mass of carbon dioxide absorbed in the sodium hydroxide in the outlet circuit becomes almost equal to the CO_2_ produced by Sp5SBS, Sp10SBS, and Sp20SBS. The Sp30SBS composite produces more carbon dioxide than all the other samples. This behavior may be explained by a faster and easier biodegradation of proteins contained in the spirulina biomass, relative to other biopolymers. Also, one may appreciate that the decomposition of proteins into amino acids leads to an attractive source of nutrients for the microbiome of the biodegradation media and may cause higher biomass growth [28,29].

### 3.2. Physical Properties Testing of Polymer Composites

**Figure 3:**
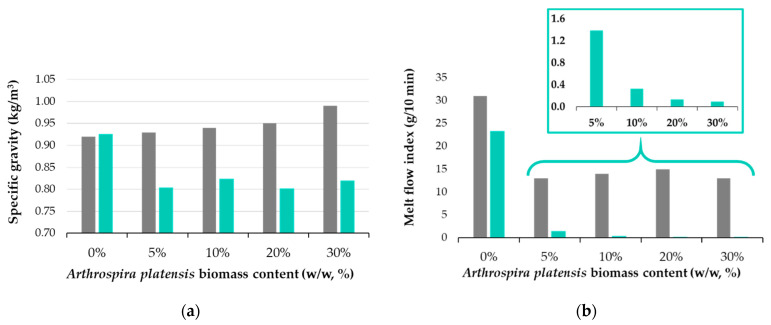
Physical properties of polymer composites: (**a**) specific gravity and (**b**) melt flow index for initial samples (grey bar) compared with samples after 180 days of biodegradation in industrial composting conditions (green bar).

Figure 3 shows the physical test results for the polymer composite samples before and after 180 days of biodegradability testing. The polymeric composite without incorporated biomass does not show changes in specific gravity (~0.92 kg/m^3^). At the same time, it is observed that the initial samples containing spirulina register a slight increase in density due to the addition of biomass in the polymer matrix, from 0.93 kg/m^3^ to 0.99 kg/m^3^ for the composite with 30% added spirulina. The increase is linear and proportional to the percentage of biomass added to the polymer. The samples subjected to the biodegradation test have lower densities compared to the initial composite (0.78–0.83 kg/m^3^); the highest reduction in density was recorded for the Sp30SBS composite samples (0.17 kg/m^3^).

The melt flow index (MFI) values are presented in Figure 3b, and it is observed that for all situations the addition of biomass leads to composites with reduced flowability. The reduction in MFI values could be related to several factors such as the interaction between spirulina biomass and the SBS matrix, changes in the rheological properties of the composite, or the formation of new molecular structures. The presence of macroconstituents like proteins, carbohydrates, and lipids in spirulina biomass can influence the melt flow properties of the composite material by the formation of noncovalent intramolecular bonds between these macroconstituents and the polymer matrix (SBS in this case) [30]. These interactions can lead to alterations in the macromolecular chain structure of the polymer and consequently can affect various properties of the polymer, including its viscosity and flow behavior during processing. It was also noticed that the percentage of biomass fillers does not influence the MFI value for the initial samples, that is, before biodegradation in industrial composting conditions (58 °C and 50% humidity).

Figure 3b illustrates the changes in the melt flow index (MFI) of the SBS composites pre- and post-industrial composting test, providing information about the impact of spirulina biomass on polymer properties during degradation in industrial composting conditions. Notably, the results reveal a significant decrease in the MFI for biodegraded polymer samples containing spirulina. This observed decrease, from 31.0 ± 0.5 g/10 min to approximately 23.3 ± 0.6 g/10 min in the SBSC composite, suggests material alterations likely attributed to the partial biodegradation of the polymer matrix itself. Moreover, the reduction in the MFI post-biodegradation, particularly evident in spirulina-loaded samples, correlates with the biomass content, indicating a lower MFI at higher biomass loadings in the composites (Figure 3b), with the most significant reduction observed in samples with 30% spirulina biomass. These experimental findings show that substantial changes occur in the material and chemical structure of the polymer matrix under the studied industrial composting conditions.

The variations in the melt flow index (MFI) observed in this study may be attributed to several factors. These include the possibility of material cross-linking, the breakdown of the bonds responsible for polymer elasticity, or the formation of pores within the polymer matrix. Cross-linking may occur between degraded polymer molecules and short-chain molecules resulting from spirulina biomass biodegradation. Consequently, these newly formed bonds may restrict polymer segment movement, decreasing flowability [31,32]. Another contributing factor could be the loss of plasticity generated by the degradation of polymer components essential for elasticity, resulting in a more rigid material. In SBS-based polymers, polybutadiene segments that are responsible for elasticity contain unsaturated bonds susceptible to reactions with biodegradation by-products [32]. Furthermore, biodegradation-induced pore formation, particularly in biomass-loaded samples, may create a porous, sponge-like material that does not flow, as was found during MFI testing. This explanation aligns well with findings from specific gravity tests, which indicate a decrease in specific gravity in biomass-loaded samples, as well as with SEM microscopy tests, as will be detailed below.

### 3.3. FTIR Spectroscopy

**Figure 4:**
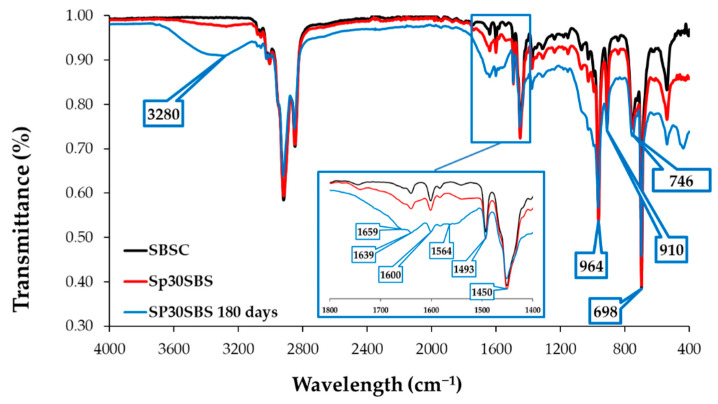
Infrared spectra of SBSC (initial, black line) compared with infrared spectra of Sp30SBS initial sample (red line) and after 180-day biodegradation test (blue line).

Figure 4 presents the infrared absorption spectra of the SBSC material (control sample) and Sp30SBS that were not subjected to the biodegradation test, in comparison with the FTIR spectrum of the Sp30SBS sample after 180 days of industrial composting testing. The three spectra were chosen for the illustration of the IR results. The spectra of the other samples containing biomass are quite similar, with small differences in the 3200–3800 cm^−1^ and 1400–1800 cm^−1^ wavenumber domain.

**Figure 5:**
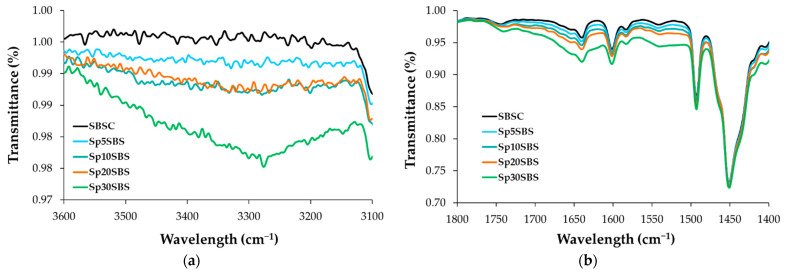
Infrared spectra of SBS-based initial composites (before biodegradation) in the regions (**a**) 3600–3100 cm^−1^ and (**b**) 1800–1400 cm^−1^.

The spectral analysis of composite samples not exposed to the biodegradation test reveals absorption bands that fall between those of the SBSC polymer and the composite containing 30% spirulina biomass, as depicted in Figure 5.

A comparable trend is observed in the biodegraded samples, with the highest absorbance peak associated with the Sp30SBS sample after 180 days of composting. It is noticeable that the absorption band in the region 3800–3200 cm^−1^ is larger for biodegraded samples after 180 days of industrial composting. This could be correlated with the increasing number of O-H and N-H stretching vibrations as a result of chemical structure changes during the biodegradation test (Figure 5a).

Also, the infrared (IR) spectra analysis of polymer samples containing SBS and spirulina biomass after biodegradation tests showed that the absorption bands were modified in the region between 1800 and 1400 cm^−1^. This modification indicates changes in the chemical composition or structure of the polymer samples, likely due to the composting process. Specific peaks at 1564 cm^−1^, 1639 cm^−1^, and 1659 cm^−1^ were recorded for the composted samples. These peaks are characteristic of amide bonds in protein structures, suggesting the presence or modification of proteinaceous material (such as spirulina) due to composting [33]. The absorption bands at 746, 910, and 964 cm^−1^ are characteristic of cis-1,4, 1,2, and trans-1,4 butadiene units, respectively [34].

In Figure 4, the absorption peaks that appear in the 1000–650 cm^−1^ wavenumber region of the IR spectra correspond to the C-H bending vibrations of C with sp^2^ hybridization. The band that appears at 746 cm^−1^ is correlated with the presence of monosubstituted alkenes, while the band at 910 cm^−1^ shows the vibrations of disubstituted alkenes. The band at 1450 cm^−1^ is correlated with C-H (C sp^3^) scissoring vibrations, while the -C–H stretching vibrations of C sp^3^ arise at 2920 and 2850 cm^−1^ [35].

The absorption band at 964 cm^−1^ indicates the presence of the 1,4-trans structure of polybutadiene in the polymer matrix. The presence of this band and its intensity could be thus correlated with the polybutadiene content in the SBS copolymer. Another IR feature characteristic of SBS polymers is the absorption band at 698 cm^−1^ that arises as a result of vibrations associated with monosubstituted benzene rings, which is a key structural component of polystyrene blocks from the polymer matrix.

Related to these absorption bands, Canto et al. reported the use of the ratio of absorbance at 964 cm^−1^ relative to 698 cm^−1^ to provide a method of quantifying the polybutadiene/polystyrene ratio in the copolymer. This report can be useful to qualitatively assess the balance between the elastic polybutadiene units and the rigid polystyrene units in the SBS composite [36].

**Figure 6:**
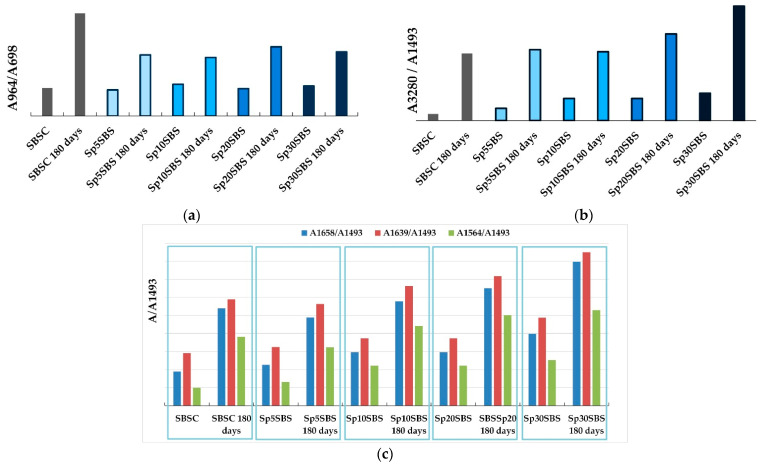
Comparison of absorbance ratios: (**a**) A964 cm^−1^/A698 cm^−1^, (**b**) A 3280 cm^−1^/A 1493 cm^−1^, and (**c**) A 1564 cm^−1^/A 1493 cm^−1^, A 1639 cm^−1^/A 1493 cm^−1^, and A 1658 cm^−1^/A 1493 cm^−1^, calculated for initial samples and samples that underwent the industrial composting test.

Figure 6a shows the ratio of the absorbance values measured at 964 cm^−1^ and absorbance values measured at 698 cm^−1^. It was observed that the initial samples show similar absorbance ratios, regardless of the added biomass percentage, which shows that the addition of biogenic material does not influence the ratio between polybutadiene and styrene in the composites that were not subjected to the biodegradation test. The situation changes notably for the samples that underwent the composting test. The ratio A964/A698 changes in favor of the butadiene component, indicating that the biodegradation process affects the two blocks in the SBS structure differently. Thus, it can be concluded that the polymer chains are predominantly attacked at the aromatic nucleus through possible substitution reactions that reduce the number of monosubstituted benzene groups.

The absorption bands at 1493 cm^−1^ and 1600 cm^−1^ are specific to C-C stretching aromatic ring mode vibrations and are referred to as reference peaks for polystyrene compounds [37]. For the studied materials, it was observed that the position of the two peaks remains unchanged, but the intensity of the band at 1600 cm^−1^ is influenced by the broad absorption band that appears in the spectra of biodegraded samples in the 1700–1500 cm^−1^ wavenumber region. Therefore, the absorption band at 1493 cm^−1^ was set as the reference band for comparing the spectra of all compounds before and after biodegradation because its position, width, and intensity remained approximately the same for all tested samples, and thus it provides the characteristics needed for a reference point.

The ratios between the absorbance measured at 3280 cm^−1^ and the one measured at 1493 cm^−1^ may provide qualitative indications about the way in which the biodegradation process unfolds in the polymer structure. Thus, it was observed that the spectra of initial samples showed a slight increase due to the addition of biomass, which is intuitive because more loaded biomass causes more macroconstituents with O-H and N-H bonds (carbohydrates, proteins, lipids, etc.) in the composite polymer matrix. The results that stand out are the absorbance ratios A3280/A1493 for the biodegraded samples, which indicate an increase in the number of O-H and N-H vibrations in the polymer structure. This indicates that processes leading to the modification of the biodegraded composite structure are occurring during the industrial composting tests (Figure 6b). It was observed that the greatest increase in the absorbance ratio was recorded for the composite with 30% spirulina biomass content, indicating that the microalgae biomass could be considered as a promoter of the biodegradation process.

Another region that attracts attention in the spectra of the composites subjected to the composting test is the area 1500–1800 cm^−1^, where an increase in the absorbance ratios for peaks at 1564, 1639, and 1658 cm^−1^ relative to the reference peak at 1493 cm^−1^ was observed. Figure 6c shows the evolution of absorbance ratios calculated relative to the absorbance peak recorded at 1493 cm^−1^. The trend presented in the case of the A3280/A1493 ratio is also preserved for these ratios (Figure 6b,c). This variation recorded in the area of double bonds (C=C, C=O, C=N), correlated with the increase in the ratios in the O-H and N-H stretch vibration area, can be interpreted by the appearance of new bonds in the polymeric material. The position of the bands that change their intensity (1564, 1639, and 1658 cm^−1^) may indicate the possible increase in the number of peptide bonds in the polymeric material.

### 3.4. SEM Analysis

**Figure 7:**
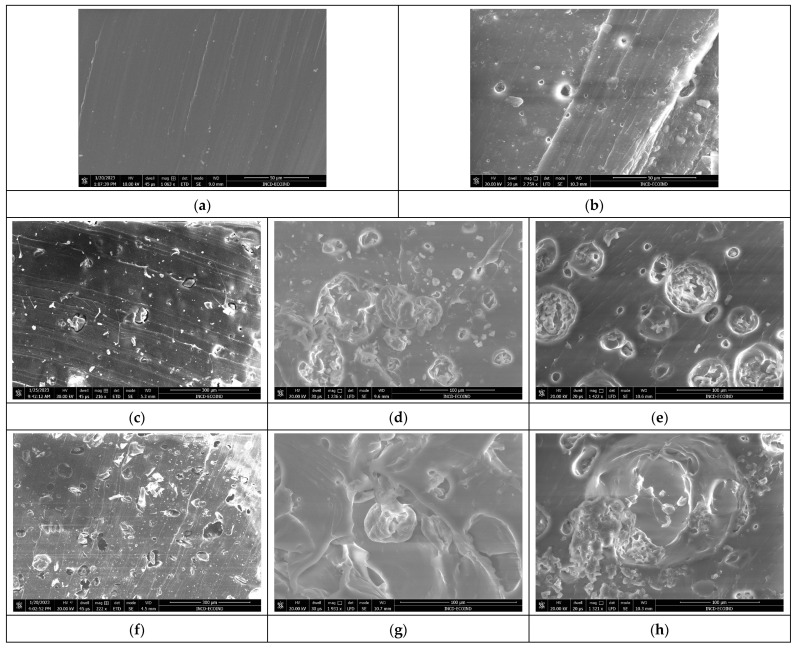

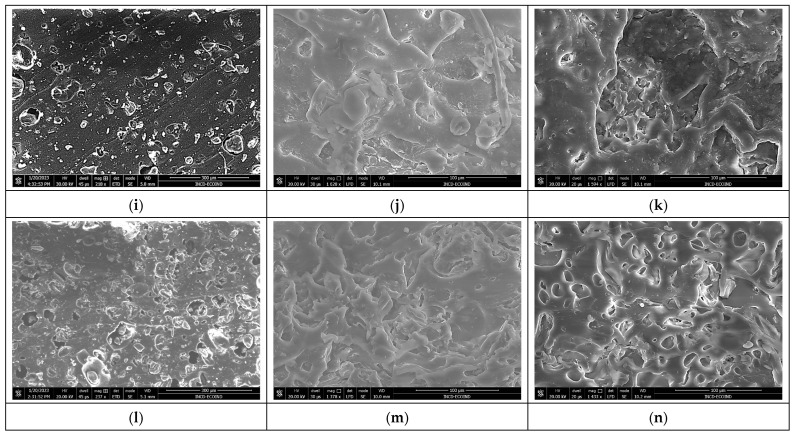
SEM images of polymer SBS composites before and after biodegradation: (**a**) SBSC initial sample, (**b**) SBSC after 180 days, and SBS–*Arthrospira platensis* composites, (**c**) Sp5SBS initial sample, (**d**) Sp5SBS after 100 days, (**e**) Sp5SBS after 180 days, (**f**) Sp10SBS initial sample, (**g**) Sp10SBS after 100 days, (**h**) Sp10SBS after 180 days, (**i**) Sp20SBS initial sample, (**j**) Sp20SBS after 100 days, (**k**) Sp20SBS after 180 days, (**l**) Sp30SBS initial sample, (**m**) Sp30SBS after 100 days, and (**n**) Sp30SBS after 180 days.

The images presented in Figure 7a–n show the surface morphology of the composite polymer materials before and after biodegradation. The images were obtained for the surfaces of the freshly cut samples.

The images shown in Figure 7 show that all the studied polymers are affected by the biodegradation tests. The composite that does not contain incorporated biomass is the least affected by the composting conditions, as seen in Figure 7a,b, while the composites with incorporated biomass exhibit significant degradation. The samples subjected to the composting test show substantial surface changes regardless of the added biomass concentration. An important detail is noticeable in the freshly cut Sp5SBS and Sp10SBS samples after 100 days, which show the presence of spirulina globules in the polymer matrix (Figure 7d,g). This shows that some biomass remains non-degraded in the polymer matrix.

Analyzing the surfaces shown in the images in Figure 7, it can be concluded that biomass undergoes the same degradation process as the polymer but at a higher speed.

In the polymer composites analyzed after 180 days, the appearance of grooves and craters in the polymer mass was observed. The higher the biomass content, the higher the number of voids created in the polymer matrix (sponge-like material).

### 3.5. DSC Analysis

Differential Scanning Calorimetry (DSC) analysis for samples heated in air was used to test the thermal properties of materials before and after the composting test. In the context of the present study on SBS composites with *Arthrospira platensis* biomass, DSC analysis can provide further insights into the behavior of the polymer throughout the composting process.

DSC analysis for tested samples can track any shifts in thermal properties as the polymer undergoes degradation. This could include changes in the Tg (glass transition temperature) or shifts in heat flow associated with degradation reactions. These changes can indicate alterations in the molecular structure or composition of the polymer that appeared because of biodegradation time and conditions. Integrating DSC data with findings from physical–mechanical testing and FTIR and SEM analysis allows for a comprehensive understanding of the degradation mechanisms that occur in the SBS composites.

**Figure 8:**
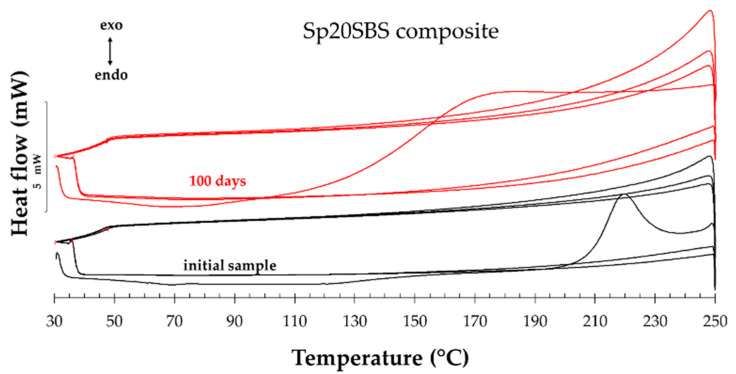
Example of three heating–cooling cycles of DSC analysis.

Figure 8 presents an example (Sp20SBS composite) of the composite before and after the composting test that was subjected to three heating–cooling cycles of the DSC test procedure. In the plot, the vertical Oy axis shows the heat flow normalized with the sample mass. It was noticed, for all samples tested, that when performing the DSC test (samples heated and cooled in the air in the 30 °C … 250 °C range), a first heating stage is recorded that shows thermal effects associated with the glass transition, desorption/volatilization of chemical compounds, and a possible cross-linking, which disappear while reheating in the second and third cycles.

In the second and third heating cycles, the disappearance of these effects could indicate that the material’s thermal behavior becomes consistent and repeatable after the initial cycle. This indicates that the material undergoes a degree of thermal conditioning or transformation during the initial heating cycle. This stabilization could be due to the removal of volatile compounds or the completion of any chemical reactions that occurred during the first cycle.

The comparison of the thermal effects in the 2nd and 3rd heating cycles did not show significant differences between the tested materials, and therefore, we compared the first heating stage of all the samples subjected to the composting test because the recorded values of the DSC analysis indicate differences in behavior between the samples collected during the testing.

**Figure 9:**
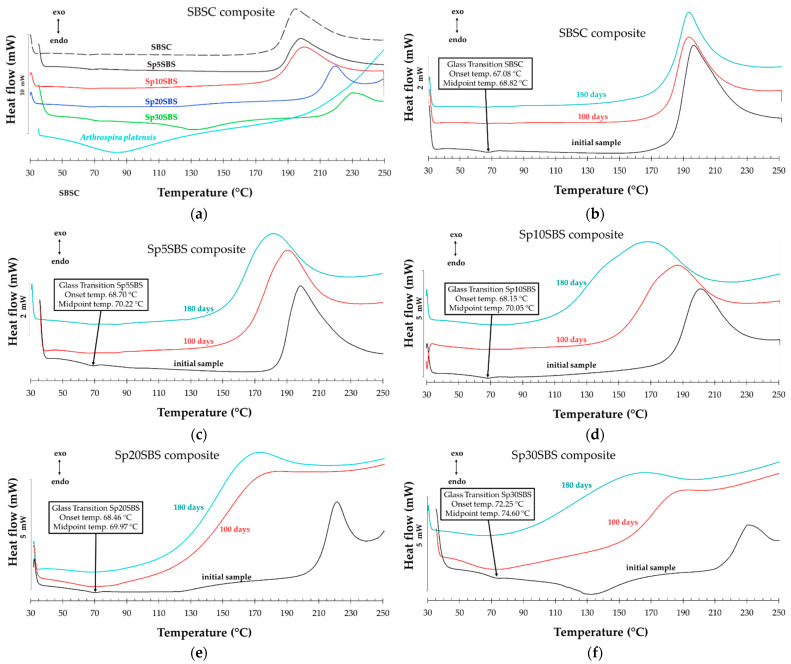
DSC thermograms for (**a**) SBS-based initial composites and comparative DSC curves for composites before (black lines) and after biodegradation for 100 days (red lines) and 180 days (blue lines) for (**b**) SBSC, (**c**) Sp5SBS, (**d**) Sp10SBS, (**e**) Sp20SBS, and (**f**) Sp30SBS composites.

Figure 9 shows the DSC analysis for the composite samples before and after 100 days and 180 days of industrial composting. Figure 9a shows the DSC curves for the initial samples before being subjected to the biodegradation test. It was observed that all the polymeric materials present a glass transition whose onset temperature increases slightly for the composites containing spirulina biomass compared to the composite without loaded biomass (~67 °C). The greatest increase in onset Tg is observed in the compound with 30% spirulina. The glass transition temperature is a critical parameter, a turning point, where the material transforms from a rigid, glassy state to a more flexible, rubbery state. At temperatures below the Tg, the polymer chains are typically immobilized through intermolecular bonds, leading to an ordered structure. As the temperature increases and reaches values close to the Tg, the energy within the polymer chains begins to match or exceed the energy of intermolecular bonds, thus allowing for increased molecular mobility.

The increase in the Tg for the composite with spirulina biomass content suggests that the loaded biomass interacts with the polymer chains through intermolecular forces, leading to a more ordered structure. This interaction likely influences the mobility of the polymer chains, increasing the Tg. Furthermore, when the polymer undergoes biodegradation, the DSC thermograms show the disappearance of the glass transition (Figure 9b–f). This is explained as related to the effect of biodegradation that typically breaks down the polymer chains, leading to a loss of order and rigidity [38].

**Figure 10:**
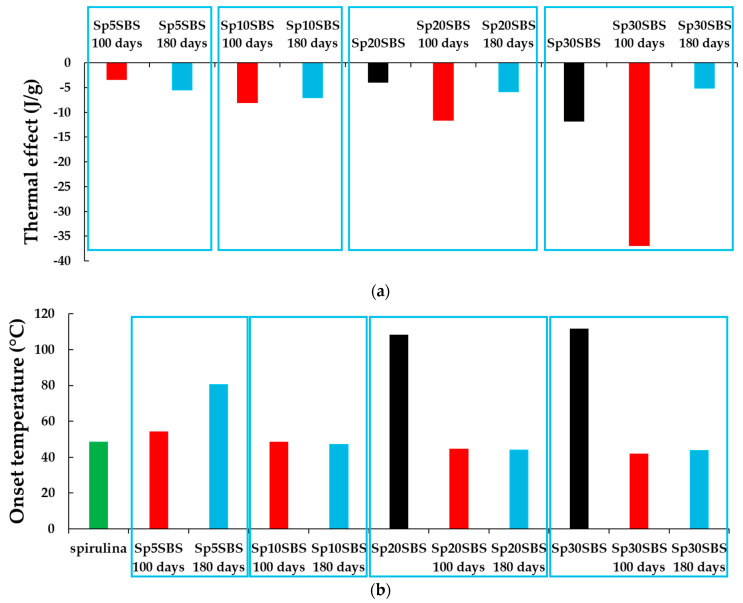
Graphical representation of (**a**) the normalized endothermal effect (J/g) registered in the 50–120 °C domain and (**b**) the onset temperature variations in the endothermal effect, observed in the first cycle of heating for SBS composites filled with *Arthrospira platensis* biomass.

When the initial composite samples are heated above the Tg, an endothermic thermal effect appears over 100 degrees Celsius in the samples containing 20% and 30% spirulina biomass, whose value normalized to the substance mass (J/g) increases with the percentage of biomass added (Figure 10a,b—black bar). This suggests that the biomass content may be contributing to the overall energy absorption during these thermal transitions.

The large endothermic effect indicates heat absorption, which can be attributed to processes like desorption, sublimation, or dehydration. The spirulina biomass may contain components that undergo specific chemical reactions or decomposition processes at elevated temperatures, leading to the observed endothermic effect.

In the samples subjected to the biodegradation tests, it can be observed that when testing composites with spirulina loaded in the matrix, the DSC curves show a wide thermal effect that starts at over 40 °C (Figure 10b) which may be associated with the removal of water from the degraded material. In the case of the 100-day-biodegradation-tested samples, the calculated thermal effect (Figure 10b) is higher than the one calculated for the samples subjected to the composting test for 180 days and is proportional to the added biomass content. The results are consistent with the SEM images of the sectioned surfaces for the samples that were subjected to composting for 100 days. The remaining biomass in the polymer can keep water in the polymer matrix which opens more channels for the absorption of water molecules due to the degradation of the polymer matrix. After 180 days of composting tests, the amount of biomass trapped in the polymer becomes smaller due to the increase in the spaces through which chemical compounds can diffuse due to the alteration in the polymer structure.

**Figure 11:**
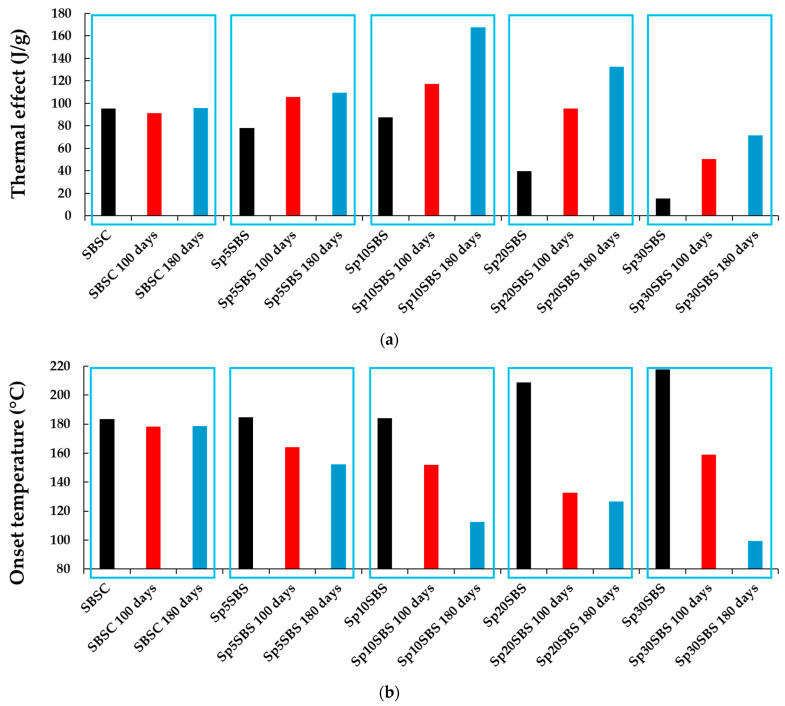
Graphical representation of the (**a**) normalized exothermal effect (J/g) and (**b**) onset temperature (°C) registered in the first cycle of heating of SBS-based composites with different loading % of *Arthrospira platensis* biomass.

The exothermic effect presented by the composite samples before and after the biodegradation test is different depending on the added biomass content and the number of days of industrial composting. The least influenced by biodegradation was the SBSC sample, which does not show significant changes in the thermal effect (Figure 11a) and onset temperature (Figure 11b). The samples that contained added spirulina biomass show an increase in the exothermic thermal effect and a decrease in the onset temperature with the increase in the number of composting days.

The broadening of the DSC peak is explained by multiple overlapping processes and different reaction kinetics. The sample may undergo multiple overlapping exothermic processes, each contributing to the overall heat release. These processes might have different activation energies or occur at slightly different temperatures, leading to a broadened peak. This indicates that the biodegradation process induces changes in the biomass and polymer matrix resulting in a wide range of degradation products that could interact at higher temperatures.

## 4. Discussion

The experimental findings confirm that the incorporation of *Arthrospira platensis* biomass into polymer composites can enhance the biodegradation process, primarily due to the macroconstituent composition of the algal biomass. Spirulina is a type of cyanobacteria rich in proteins, carbohydrates, lipids, and other nutrients, making it a valuable biomass source for various applications, including biodegradable polymer composite production. Furthermore, spirulina is a renewable resource that can be cultivated relatively easily in controlled environments like ponds or bioreactors.

Some studies examined the utilization of microalgae biomass rather than isolated components from microalgal biomass in conjunction with polyethylene (PE) and polypropylene (PP) blends. The integration of microalgae into polymers has predominantly focused on enhancing mechanical properties. However, recent findings, as highlighted by Mateescu et al., emphasize the stabilizing effects of microalgae in PP. According to these authors, the microalgal biomass served as a protective agent against PP degradation, primarily attributed to polyphenols present in microalgae [23]. Additionally, the use of polyurethane with microalgae can lead to superior mechanical properties. This underscores the growing interest in developing bioplastics incorporating microalgae as a promising alternative in the realm of eco-friendly materials, as highlighted by Kim et al. [39]. Another study by Parin et al. reported effective antibacterial thermoplastic polyurethane/polycaprolacton (TPU/PCL) composite nanofibers loaded with spirulina biomass by the electrospinning method for wound care applications [40]. Spirulina biomass was also used for polymer blends with PLA, as reported by Liao et al. Mechanical testing revealed that the elastic properties of PLA were retained in the bio-composites with 23% of spirulina biomass in the polymer matrix [20].

Spirulina biomass is organic and biodegradable, which could contribute to the development of biodegradable polymers. In the present study, it was shown that, initially, spirulina biomass produces more carbon dioxide within the first 100 days of the composting test. However, after this period, the carbon dioxide evolved trapped by NaOH bubblers becomes almost equal to the CO_2_ produced by the biodegradation of the composites containing spirulina biomass at various concentrations (Sp5SBS, Sp10SBS, and Sp20SBS). Interestingly, the composite with a higher concentration of spirulina biomass (Sp30SBS) produces more carbon dioxide than all the other composites. This behavior suggests that the biodegradability of spirulina biomass facilitates a faster degradation process. Figure 2 indicates that spirulina biomass degrades faster than the polymer component of the composites, which is intuitive considering the chemical composition of both materials. Additionally, after 180 days, the amount of CO_2_ produced by the composites with embedded biomass exceeded that of the reference composite SBSC or the algal biomass alone, when samples with identical masses were used. This observation further supports the concept that spirulina biomass promotes the biodegradation of the polymer.

The difference in the degradation rate is also supported by results from the Differential Scanning Calorimetry (DSC) and Scanning Electron Microscope (SEM) analyses. SEM images show that microalgae granules are still present in the polymer matrix after 100 days, whereas they disappear in samples subjected to the composting test for 180 days. Moreover, DSC reveals an endothermic thermal effect associated with the desorption and volatilization of water molecules from the polymer matrix in samples with a high content of algal biomass, confirming the presence of the hydrophilic biomass in the polymer matrix. After 180 days, DSC confirmed that the effect decreases (Figure 11a), indicating that a large amount of biomass diffused from the material. This is possible because the polymer matrix degrades and the holes in the material become bigger (conclusion confirmed by the SEM and specific gravity testing). Another reason for biomass migration from the polymer is related to the biodegradation of biomass that produces molecules with smaller sizes that could leave the polymer matrix.

The changes in the melt flow index (MFI) observed in the SBS composites pre- and post-industrial composting test are illustrated in Figure 3b. The significant decrease in the MFI for all biodegraded polymer samples suggests material alterations likely attributed to partial biodegradation of the polymer matrix itself. For instance, in the SBSC composite, the MFI decreases from 31.0 ± 0.5 g/10 min to approximately 23.3 ± 0.6 g/10 min post-biodegradation. This reduction in the MFI post-biodegradation is more evident for spirulina-loaded composites, and it correlates with biomass content, showing a lower MFI with higher biomass loading. The most significant reduction is observed in samples with 30% spirulina biomass.

Several factors which contribute to the variations in the MFI observed under composting conditions are considered: cross-linking between degraded polymer molecules and short-chain molecules resulting from biomass biodegradation, the breakdown of bonds in the polybutadiene segments, and the formation of a sponge-like structure that does not flow during MFI testing.

The cross-linking hypothesis is supported by the FTIR and DSC analysis of the samples. The IR spectra recorded for samples before and after biodegradation could indicate the formation of new peptide bonds in the structure of the material. This conclusion is supported by the analysis of IR absorption bands in the regions 3800–3200 cm^−1^ and 1800–1500 cm^−1^ (N-H and amide C=O vibrations). Another finding offered by the analysis of IR data is that the primary target of the biodegradation process is the styrene block of the SBS composite, as presented by the evolution of the A964/A698 ratio (Figure 6a).

The analysis of the peak associated with exothermic thermal effects in DSC analysis provides further insights into the chemical transformations occurring within the polymer matrix. The disappearance of the exothermic peak when tested in successive heating–cooling cycles in the range of 30–250 °C indicates irreversible chemical transformations with a positive energy balance. This suggests that the polymer undergoes permanent changes likely due to the reticulation or cross-linking of the material, which occurs differently in each sample. The heating process may induce a structural rearrangement or cross-linking within the polymer, influenced by the presence of spirulina biomass and biodegradation products.

An important characteristic observed in the DSC analysis is the broadening of the recorded exothermic peak. This broadening can be attributed to the development of complex chemical reactions, likely stemming from the interactions between the polymer, the biomass, and the biodegradation products of the biomass. These interactions lead to the occurrence of some intricate chemical processes within the polymer matrix, contributing to the observed broadened exothermic peak.

The results presented in this study are in good agreement with findings on the degradation of styrene compounds. Previous research has shown that various microorganisms can metabolize styrene aerobically as their sole source of carbon and energy [41,42,43,44]. Under aerobic conditions, styrene–butadiene–styrene (SBS) may undergo metabolism by oxidizing the C=C bond in polybutadiene segments. Additionally, earlier studies have reported the oxidation of the aromatic ring [45,46,47,48]. Monooxygenases or aromatic ring hydroxylases are promising candidates for cleaving the aromatic ring of the styrene block. However, the specific degradation pathway and the enzymes involved in this process have not yet been fully elucidated. Direct attack on the aromatic ring leads to the formation of styrene cis-glycol, followed by 3-vinylcatechol. Subsequently, 3-vinylcatechol undergoes ring-cleavage, producing muconic acid derivatives [41,42]. Also, it was demonstrated that under abiotic conditions, photo- or thermo-oxidation processes generate multiple compounds, primarily short, oxidized chains resulting from main-chain scissions, containing high levels of unsaturation and carbonylated functions. These short chains can be released into the water phase, potentially serving as substrates for microorganisms or as new cross-linking agents within the polymer matrix [49]. In a study by Olejnik et al., it was shown that within a single cycle of bacterial culture, there was an average reduction of over 4% in rubber waste mass [50]. Conversely, spirulina biomass undergoes degradation in industrial composting conditions [51], resulting in a variety of degradation compounds. These compounds have the potential to establish new bonds with the products of SBS degradation. Taken together, the experimental results confirm the formation of peptide bonds between the degradation products of biomass proteins and the products arising from the degradation and oxidation of the aromatic ring.

## 5. Conclusions

The evaluation of SBS composites loaded with *Arthrospira platensis* biomass in an industrial composting test provides information about the feasibility of utilizing algae as a filler material in thermoplastic composites and its impact on biodegradation and physical properties. The physical–chemical testing revealed a decrease in the specific gravity of the samples after the industrial composting test, indicating a reduction in material density likely due to degradation and loss of mass during composting. Additionally, the measurement of the melt flow index (MFI) showed a significant decrease post-biodegradation, suggesting limited recyclability or reusability of degraded materials through conventional thermoplastic processing techniques. This decrease in the MFI indicates alterations in material flow properties, which could affect processing and application.

Furthermore, the results from FTIR, SEM, and DSC analysis indicated that the properties of the composites undergoing the composting process were influenced by the content of microalgae biomass added to the composite. Specifically, the physical–chemical changes observed, along with data from FTIR, SEM, and DSC analysis, suggest that the biodegraded polymer structure exhibits increased cross-linking and higher porosity in samples with higher biomass content incorporated into the polymer matrix. The addition of spirulina biomass affects not only the biodegradation rate but also significantly impacts the physical properties of the composite material during degradation.

The results presented in this study are in good agreement with findings on the degradation of styrene compounds. Under aerobic conditions, styrene–butadiene–styrene (SBS) may undergo the oxidation of the C=C bond in polybutadiene segments and the oxidation of the aromatic ring. A direct attack on the aromatic ring leads to the formation of muconic acid derivatives that can establish new bonds with spirulina biomass degradation products. The experimental results confirm the formation of peptide bonds between the degradation products of protein-rich biomass of spirulina and the products arising from the degradation and oxidation of the aromatic ring. The behavior of the composites with spirulina during the industrial composting experiment suggests not only their degradation but also the modification of the materials at the structural level, which significantly influences their physical–mechanical properties.

## Data Availability

The data presented in this study are available within the present article.
